# circStrn3 is involved in bone cancer pain regulation in a rat model

**DOI:** 10.1093/abbs/gmaa018

**Published:** 2020-05-12

**Authors:** Yiwen Zhang, Xiaoxia Zhang, Zumin Xing, Shuyi Tang, Hanwen Chen, Zhongqi Zhang, Jiyuan Li, Yalan Li

**Affiliations:** 1 Department of Anesthesiology, The First Affiliated Hospital of Jinan University, Guangzhou 510632, China; 2 Department of Anesthesiology, Shunde Hospital of Southern Medical University (The First People’s Hospital of Shunde Foshan), Foshan 528308, China

**Keywords:** bone cancer pain, differentially expressed genes, circRNAs, circStrn3

## Abstract

Bone cancer pain (BCP) is a common chronic pain that is caused by a primary or metastatic bone tumor. More detailed molecular mechanisms of BCP are warranted. In this study, we established a BCP rat model. The von Frey hair test, body weight, and hematoxylin and eosin staining were employed. We screened differentially expressed circRNAs (DECs) between the BCP group and sham group. The results revealed that 850 DECs were significantly up-regulated and 644 DECs were significantly down-regulated in the BCP group. Furthermore, we identified 1177 differentially expressed genes (DEGs) significantly up-regulated and 565 DEGs significantly down-regulated in the BCP group. Gene Ontology annotation of all 1742 DEGs revealed that biological regulation of metabolic processes, cellular processes, and binding were the top enriched terms. For Kyoto Encyclopedia of Genes and Genomes analysis, phagosome, HTLV-I infection, proteoglycans in cancer, and herpes simplex infection were significantly enriched in this study. In addition, we identified four selected circRNAs, chr6:72418120|72430205, chr20:7561057|7573740, chr18:69943105|69944476, and chr5:167516581|167558250, by quantitative real time PCR. chr6:72418120|72430205 (circStrn3) was selected for further study based on expression level and the circRNA–miRNA–mRNA network table. Western blot analysis suggested that knockdown of circStrn3 could effectively induce Walker 256 cell apoptosis. In summary, our study provided a more in-depth understanding of the molecular mechanisms of BCP.

## Introduction

Bone cancer pain (BCP) is a chronic pain caused by a primary or metastatic bone tumor. Clinically, the incidence of metastatic bone tumors is about 35–40 times higher than that of primary malignant bone tumors. Therefore, most malignant bone tumors are caused by bone metastasis from advanced cancer [1]. According to the World Health Organization, there are 10 million new cancer patients in the world annually and China has an annual increase of about 1.8 million [2]. The incidence of bone metastases in malignant tumors is 32.5% [3], of which more than 90% of bone metastases are derived from five tumor types, including breast cancer, prostate cancer, lung cancer, thyroid cancer, and kidney cancer [4–6]. When tumor cells, such as osteosarcoma cancer cells and breast cancer cells, invade into the bone, proliferation of tumors can lead to a variety of bone structural changes, including osteolysis, nonbone tissue formation, hypercalcemia, and inflammatory factor release [7]. These changes stimulate stromal cells to secrete the receptor RANKL, which promotes osteoclast proliferation and hypertrophy through the RANKL/RANK signaling pathway [8]. According to statistics, ~70% of patients with bone metastases have various degrees of chronic pain [9]. Although many drugs are available to treat BCP, such as antidepressants, anticonvulsants, and opioids, there may be various side effects such as nausea, dizziness, arrhythmia, and hyperalgesia, which lead to restrictions on the use of these drugs [10]. Meanwhile, there is still a lack of understanding of the mechanism of BCP and there are also limitations of existing clinical treatment measures. For example, ~50% of cancer patients are not effectively controlled in the symptoms of BCP [11,12]. Therefore, an in-depth study of the mechanism of BCP is absolutely critical.

Although circRNAs were discovered more than 30 years ago, they were once thought to be the ‘garbage’ and ‘noise’ of gene transcription. Therefore, these molecules were once considered to have no specific biological functions and did not receive widespread attention from researchers. Recent studies on circRNAs have led to the recognition that the transcripts of many human genes can be reversed by nonlinear reverse cleavage [13] or by gene rearrangement [14]. Meanwhile, there are many unique characteristics, including an abundance of all shear transcripts that are structurally stable and sequence-conserved, with tissue-specific and time-specific expressions [15,16]. Therefore, circRNAs may potentially serve as biomarkers. In multiple cancer types, previous studies have indicated that circRNAs play an important role in colony formation, proliferation, apoptosis, invasion, and metastasis, such as bladder cancer [17], liver cancer [18], colorectal cancer [19], gastric cancer [20], and pancreatic cancer [21]. The main function of circRNAs is reflected in gene expression regulation at the transcriptional or post-transcriptional level [22]. On the contrary, exon-derived circRNAs are primarily localized in the cytoplasm to block the inhibition of target genes by competitive endogenous RNA (ceRNA) binding to miRNA [23,24]. In addition, some endogenous circRNAs have the ability to translate peptides and proteins [25]. Noncoding RNAs have been shown to be involved in the regulation of BCP. For example, miR-124 can inhibit the expression of synaptopodin protein to regulate damage sensation and BCP [26]. Investigating the roles of the more stable circRNAs in the regulation of BCP may shed further light on it.

In this study, we established a rat BCP model and examined mRNA and circRNA expressions in the BCP group and sham group. Functional enrichment analysis of differentially expressed genes (DEGs) and parental gene transcription composed of circRNAs was investigated via the Kyoto Encyclopedia of Genes and Genomes (KEGG) and Gene Ontology (GO) tools. Moreover, circRNA validation was carried out by PCR, Sanger sequencing, and real-time quantitative PCR (qRT-PCR). In addition, we further explored the potential effects of knockdown of chr6:72418120|72430205 on proliferation and apoptosis of Walker 256 cells. The results obtained in this study provided detailed information of BCP.

## Materials and Methods

### Cell culture

The Walker 256 cell line was obtained from ATCC (Manassas, USA). Cells were cultured in RPMI 1640 medium (Invitrogen, Carlsbad, USA) containing 10% fetal bovine serum (Invitrogen), 100 mg/l penicillin, and 100 mg/l streptomycin at 37°C with 5% CO_2_.

### BCP rat model

Walker 256 cells were harvested at log phase. Sterile phosphate buffered solution (PBS) was used to wash cells twice. The cell density was adjusted to 1 × 10^7^ cells/ml. Female Sprague Dawley (SD) rats (60–80 g, 3–4 weeks) were purchased from the Guangdong Experimental Animal Center and fed in Forevergen Biosciences Co., Ltd (Guangzhou, China). On the sixth/seventh day, rats inoculated with Walker 256 cells (0.5 ml/dose, i.p.) showed mild ascites symptoms in the abdomen. After 9–12 days, the inoculated rats had obvious ascites symptoms. Ascites containing Walker 256 cells were drawn. An aliquot of about 2 ml of ascites was collected and centrifuged at 250 *g* for 2.5 min, and the supernatant was aspirated. Walker 256 cells were suspended in sterile PBS and centrifuged at 250 *g* for 2.5–3 min. Cells were washed twice to remove a large amount of blood cells. The cell density was adjusted to 8 × 10^7^ cells/ml with sterile PBS and placed on ice for later use. The cells of the sham operation group were boiled for 20 min and then used [27]. Female SD rats (180–210 g) were anesthetized with pentobarbital (45 mg/kg, i.p.). Afterwards, they were restrained on their backs, 75% alcohol was used to disinfect the medial skin of the left posterior tibia. Electric razor was used to remove surface hair. A 0.5-cm-long incision was made on the skin along the tibia, 0.5 cm below the tibial head. A 5-ml syringe needle was used to drill a hole perpendicular to the bone surface in the middle of the exposed tibial plateau. Then, 5 μl (4 × 10^5^ cells) of the above-mentioned Walker 256 cell suspension was injected into the marrow cavity using a sterile 25-μl microsyringe. The hole was sealed with sterile bone wax and the muscle and skin were sutured layer by layer. The animals were placed on a 37°C thermostat pad for rewarming and returned to the animal house [28]. The rats in the sham operation group were injected with boiled Walker 256 cells and subject to the same surgical procedure as described above. Ten rats were included in the sham operation group and the BCP group. The treated rats in both groups were sacrificed 21 days postsurgery [29]. The animal studies were approved by the ethics committee of the Forevergen Biosciences Animal Center.

### The von Frey hair test

The von Frey series of fibers were used to determine the threshold of response to mechanical stimuli. The rat mechanical stimulation pain threshold was expressed by the paw withdrawal threshold (PWT). Rats were placed on a metal mesh, covered with a transparent plexiglass. The rats were allowed to acclimate to the environment for 5 min. After the carding and inquiry activities of the rats disappeared, a series of standardized von Frey fibers (0.1–65 g) were used to stimulate the middle part of the hind paw from small to large until the curvature was slightly S-shaped for 6–8 s. The rats developed a rapid contraction response immediately during the stimulation time or when the von Frey fibers were removed, which was recorded as a positive reaction. The contractile response caused by physical activity was not recorded as a positive reaction. The threshold of the PWT on the surgical side of the rat was recorded as a pain threshold. The test was repeated three times, and the results were obtained from three tests.

**Table 1:** Sequence of primers used in PCR

Gene ID	Sequence (5′ →3′)	Product length (bp)
chr6:72418120|72430205-circRNA forward	GTACAGAATGGGGCACGAAT	190
chr6:72418120|72430205-circRNA reverse	CTGATTCAAAGGTGGGCATT
chr6:72418120|72430205-linear RNA forward	GCTGCTGACTTAACTGATGATCC	117
chr6:72418120|72430205-linear RNA reverse	TGTACCATCCCCTGAACTCC
chr20:7561057|7573740-circRNA forward	ACAGACGGGAGGACTGAAGA	224
chr20:7561057|7573740-circRNA reverse	TAGTGCTGCAGCTCAGGAGA
chr20:7561057|7573740-linear RNA forward	CCACACGGAGGTAGTGAAGG	161
chr20:7561057|7573740-linear RNA reverse	TAGTGCTGCAGCTCAGGAGA
chr18:69943105|69944476-circRNA forward	CGAGGAGCACGCTAAGCTAT	196
chr18:69943105|69944476-circRNA reverse	AGACAGTCCCCTTCCCTTGT
chr18:69943105|69944476-linear RNA forward	GACTGCATCGCCAGTGTCTA	222
chr18:69943105|69944476-linear RNA reverse	TTTGACGATGTTGTCGTGGT
chr5:167516581|167558250-circRNA forward	GTTCCACAGAGGATGGCTGT	218
chr5:167516581|167558250-circRNA reverse	CCGGTTCTTGATGACTGGAT
chr5:167516581|167558250-linear RNA forward	TGTTCCCGAGTTCTTGCTCT	191
chr5:167516581|167558250-linear RNA reverse	GGAAGTGGGGCCTTAGAAAG

**Figure 1:**
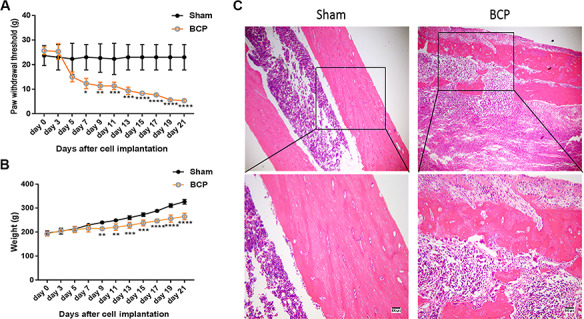
**Changes of BCP-related indexes in rat model** (A) The von Frey hair test of behavioral changes in BCP-treated rats. (B) Weight detection of BCP-treated rats. (C) H&E examination of tibia bone tissue damage in rats. Sham, control group; BCP, experimental group. ^*^P < 0.05, ^**^P < 0.01, ^***^P < 0.001, and ^****^P < 0.0001.

### Hematoxylin and eosin analysis

Hematoxylin and eosin (H&E) staining was performed as previously described [30]. The epidermis of rat was removed to expose the tibia of the rat. The tibia of the rat was cut with scissors and was fixed in 10% buffered formalin and embedded in paraffin. Sections (3 to 5 μm thick) were stained with hematoxylin (H3136; Sigma, St Louis, USA) for 10 min and then with eosin (E4382; Sigma) for 1 min to establish the diagnosis areas.

### Next generation sequencing

Ribo-Zero Gold Kit (Epicenter, Madison, USA) was used to remove ribosomal RNA from 8 μg total RNA. The total RNA without ribosomal RNA was incubated with 10 U/μg RNase R (Epicenter) at 37°C for 1 h. mRNA-Seq sample preparation kit (Illumina, San Diego, USA) was used to construct cDNA libraries according to the manufacturer’s instructions. Specifically, the 2 × 150-bp paired-end sequencing strategy was carried out on the Illumina Hiseq4000 platform. Differential expression of circRNA was identified according to the previous study [31]. In addition, the Illumina® TruSeq RNA Library Prep Kit v2 (Illumina) was used to construct the RNA-seq libraries. DEGs were obtained according to the previous method [32]. Enrichr (http://amp.pharm.mssm.edu/Enrichr/) was used to analyze GO terms enriched in DEGs. The Enrichr settings used were the same as the previous study [33]. Meanwhile, the KOBAS software was used to test the statistical enrichment of differential genes in KEGG pathways [34].

### circRNA identification

In this study, four circRNAs were selected from our high-throughput sequencing results. Eight primer pairs were designed to amplify circRNAs and their corresponding linear RNAs. The primer sequences are listed in **Table 1**. PCR reactions were performed in 20 μl volume containing 10 μl 2× GoldStar Best MasterMix (Dye) (Takara, Tokyo, Japan), 1 μl primer mixture (0.5 pmol final concentration of each), 1 μl DNA template, and 8 μl ddH_2_O. Thermal cycling conditions were as follows: 3 min at 98°C, followed by 40 cycles at 95°C for 15 s and 60°C for 25 s. The amplification products were further examined by agarose gel electrophoresis (2.0%) and Sanger sequencing. Meanwhile, qRT-PCR was performed to analyze circRNA expression in different samples. The reaction conditions were performed in 10 μl volume containing 5 μl 2× FastSYBR Mixture (Takara), 0.5 μl primer mixture (0.25 pmol final concentration of each), 1 μl DNA template, and 3.5 μl ddH_2_O. Data were collected using the ABI analytical thermal cycler. RNA expression was calculated based on a relative standard curve using the 2^−ΔΔCt^ method, representing 10-fold dilutions of the PCR products.

**Figure 2:**
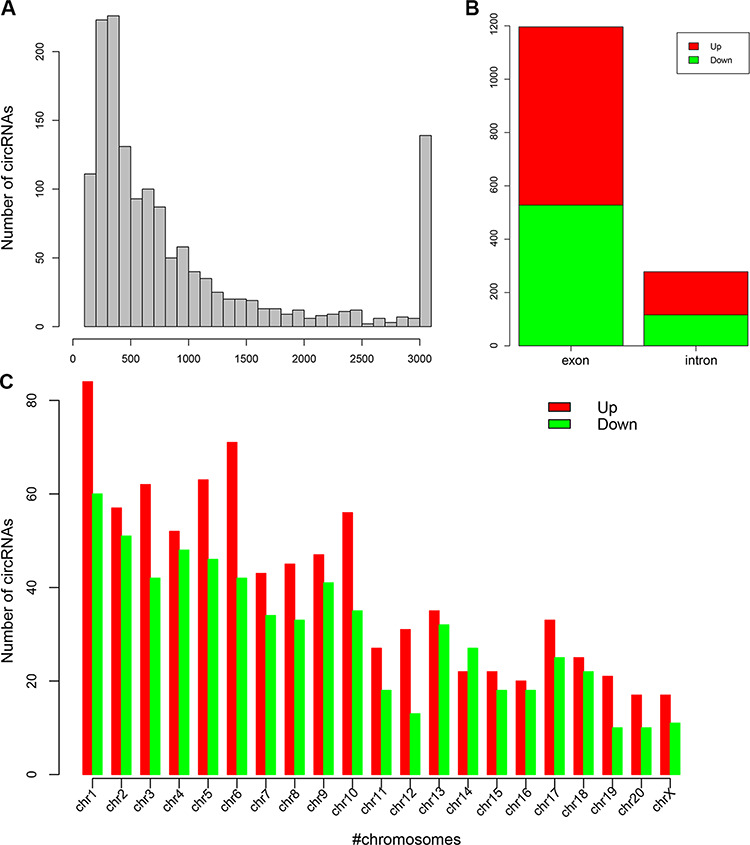
**High-throughput sequencing of circRNAs** (A) The length distribution of obtained circRNAs. (B) Exon and intron distribution of detected circRNAs. (C) Chromosomal distribution of DECs between the two groups.

**Figure 3:**
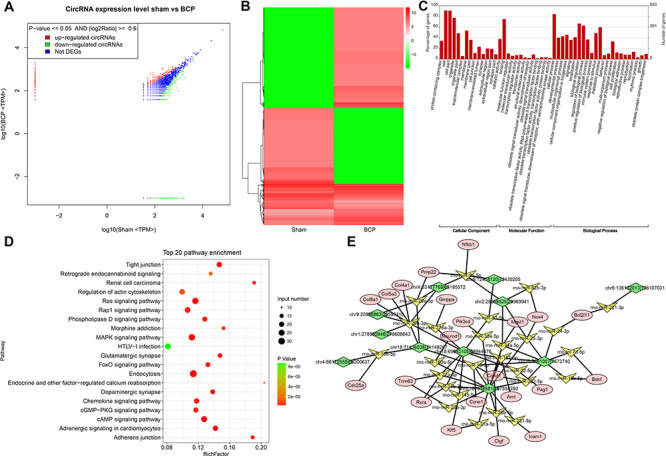
**Function analysis of the DECs between the BCP group and sham group** (A,B) DECs were visualized between the two groups by scatter plot and hierarchical clustering analysis. (C) GO analysis of the DECs. (D) KEGG analysis of DECs. (E) The circRNA–miRNA–mRNA network.

### Knockdown experiment

Double-stranded siRNAs (dsRNA) targeting the circStrn3 gene and complementary dsRNA were synthesized by Maxim Biotech (Shanghai, China). Three dsRNAs were designed for circStrn3 knockdown. The dsRNA sequences are as follows: si-circStrn3-1, 5′-GAATGGGGCACGAATTGCAT-3′ and 3′-ATGCAATTCGTGCCCCATTC-5′; si-circStrn3-2, 5′-GGTACAGAATGGGGCACGAATT-3′ and 3′-AATTCGTGCCCCATTCTGTACC-5′; si-circStrn3-3, 5′-ACAGAATGGGGCACGAATTG-3′ and 3′-CAATTCGTGCCCCATTCTGT-5′. The control siRNA sequence from a scramble sequence was 5′-UUCUCCGAACGUGUCACGUTT-3′ and 3′-TTACGTGACACGTTCGGAGAA-5′. Cells were seeded in six-well plates with 5 × 10^5^ cells per well in RPMI 1640 containing 10% fetal bovine serum without penicillin and streptomycin and cultured overnight. Transfection was carried out in OPTI-MEM serum-free medium (Gibco, Grand Island, USA) using Lipofectamine 2000 reagent (Invitrogen) and final siRNA concentration of 100 nM.

### Cell proliferation assay

Walker 256 cells were seeded in 96-well plates with 3 × 10^4^ cells perwell. Cell proliferation was detected at 24 h, 48 h, and 72 h after transfection using the Cell Counting Kit-8 (CCK-8; Dojindo, Kumamoto, Japan). All procedures were carried out according to the manufacturer’s instructions.

### Cell apoptosis

Walker 256 cells were seeded in six-well plates with 5 × 10^5^ cells per well. Cell apoptosis was detected 48 h after transfection. Cell apoptosis of differentially treated cells was analyzed using a commercial kit (C1062S; Beyotime Biotech, Shanghai, China) following the manufacturer’s protocol.

### Western blot analysis

Forty-eight hours after transfection with siRNA-NC, si-circStrn3-1, or si-circStrn3-2, total protein was isolated from Walker 256 cells using the RIPA lysis buffer (Thermo Fisher Scientific, Waltham, USA) supplemented with 1% phenylmethanesulfonyl fluoride. After being boiled with water for 5 min, the samples were subject to 12% sodium dodecylsulfate-polyacrylamide gel electrophoresis. The proteins were then transferred onto a polyvinylidene difluoride membrane (Millipore, Billerica, USA). After being blocked with 5% nonfat milk for 1 h at room temperature, the membrane was incubated with anti-bax antibody (50599-2-Ig, 1:10000; Proteintech, Rosemont, USA), anti-caspase-3 antibody (9662, 1:1000; Cell Signaling Technology, Danvers, USA), anti-Bcl-2 antibody (15071, 1:1000; Cell Signaling Technology), or anti-GAPDH antibody (600040-1-Ig, 1:8000; Proteintech) overnight at 4°C. After extensive wash, the membrane was incubated with the corresponding horseradish peroxidase-conjugated secondary antibody for 1 h at room temperature. Finally, the membrane was detected using an ECL chemiluminescence detection kit (Advansta, Menlo Park, USA) and the protein bands were analyzed with the GeneGnome 5 imaging system (Synoptics Ltd, Cambridge, UK).

### Statistical analyses

All data were expressed as the mean ± standard deviation (SD) and compared using the *t*-test method. *P* < 0.05 was considered statistically significant. All statistical analyses were performed using the SPSS for Windows version 19.

**Figure 4:**
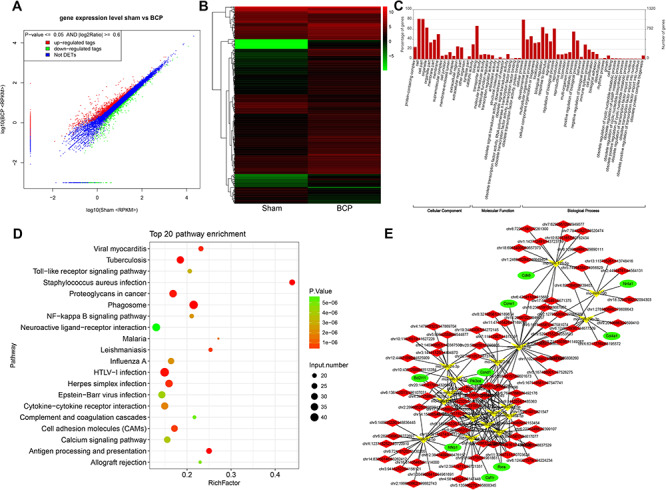
**Function analysis of the DEGs between the BCP group and sham group** (A,B) DEGs were visualized between the two groups by the scatter plot and hierarchical clustering analysis. (C) GO analysis of DEGs. (D) KEGG analysis of DEGs. (E) The mRNA–miRNA–circRNA network.

## Results

### BCP rat model

To characterize the BCP rat model, we performed the von Frey hair test, assessed body weight, and performed H&E tissue staining. The von Frey hair test showed that the pain tolerance value of PWT in the BCP group was significantly reduced compared with that in sham group from the seventh day (*P* < 0.05), suggesting that BCP rats were hypersensitive to pain (**Fig. 1A**). The body weight of BCP rats was significantly reduced compared with that in the sham group from the ninth day (*P* < 0.05), which was consistent with the von Frey hair test results (**Fig. 1B**). In addition, we examined the histopathology in the BCP group and sham group. The results clearly showed Walker 256 cell infiltration into the bone tissue and osteolysis in the rat tibia (**Fig. 1C**). These results suggested that the BCP rat model was successfully established in this study.

### Differentially expressed circRNAs between BCP and sham groups

We performed high-throughput sequencing to identify differentially expressed circRNAs (DECs) between the BCP and sham groups. The quality control analysis showed that no difference was detected in length and chromosomal distribution of circRNAs in both groups (**Fig. 2A,C**). Meanwhile, splicing origination analysis showed that most circRNAs came from exons. It was noted that the up-regulated circRNAs of the introns in the BCP group were more than that in the sham group (**Fig. 2B**). After low-quality data were filtered, the fold change method and Student’s *t*-test approach were applied to DECs analysis (**Fig. 3A,B**). In this study, we identified 850 significantly up-regulated DECs and 644 significantly down-regulated DECs in the BCP group compared with those in the sham group. GO analysis showed three main annotations, including biological processes, cellular components, and molecular functions (**Fig. 3C**). From the perspective of biological processes, cellular process, biological regulation and regulation of biological process were the top three enriched terms. From the cellular component perspective, the cell was the top significantly enriched term. From the molecular function perspective, binding and catalytic activities were the top two enriched terms. KEGG analysis showed that endocytosis was the top enriched term (**Fig. 3D**). Moreover, it is worth noting that Ras, MAPK and tight junction were also significantly enriched. In addition, we further analyzed the relationships between the top 11 DECs and their related miRNAs and mRNAs (**Fig. 3E** and **Supplementary Table S1**). The results suggested that chr6:72418120|72430205, chr20:7561057|7573740, chr18:69943105|69944476, and chr5:167516581|167558250, which were related to multiple miRNAs and mRNAs, might play important roles in BCP treatment.

### Transcriptome sequencing between BCP and sham groups

In order to further explore circRNA-related molecular mechanisms, we performed transcriptome sequencing. The fold change method and Student’s *t*-test approach were also applied to identify DEGs (**Fig. 4A,B**). In this study, we identified 1177 significantly up-regulated DEGs and 565 significantly down-regulated DEGs in the BCP group compared with those in the sham group. GO annotation showed that, from the perspective of biological processes, biological regulation, regulation of biological process and metabolic process were the top three enriched terms. From the cellular component perspective, cell and cell part were the top two enriched terms, and from the molecular function perspective, binding was the top enriched term (**Fig. 4C**). KEGG analysis showed that the phagosome was the top enriched term (**Fig. 4D**). Moreover, it is worth noting that HTLV-I infection, proteoglycans in cancer, and herpes simplex infection were also found to be significantly enriched in this study. Moreover, we further analyzed the relationship between PI3K-Akt signaling pathway and its related circRNA–miRNA–mRNA network (**Fig. 4E** and **Supplementary Table S2**). The results suggested that Nfkb1 and Ccnd1, which are related to multiple miRNAs and mRNAs, might play important roles in BCP treatment.

**Figure 5:**
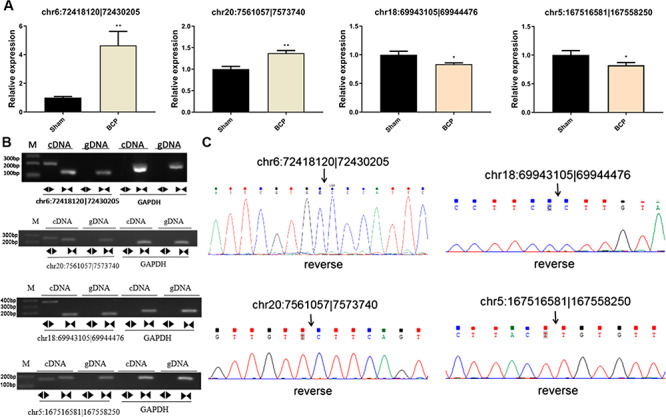
**circRNA verification and expression analysis** (A) qRT-PCR analysis of circRNA expression between the BCP group and sham group. (B) Four circRNAs were amplified using divergent and convergent primers with cDNA and gDNA of both groups. circRNA could only be amplified using cDNA templates. M: DNA molecular markers. The sizes of the two bands are 200 bp and 100 bp. (C) The head-to-tail back splicing of four circRNAs was confirmed by Sanger sequencing. The black arrow indicates the junction sequences of circRNA. ^*^P < 0.05 and ^**^P < 0.01.

### circRNA verification

In order to further confirm our sequencing results, we assessed the expression levels of chr6:72418120|72430205, chr20:7561057|7573740, chr18:69943105|69944476, and chr5:167516581|167558250 by qRT-PCR. The results showed that chr20:7561057|7573740 and chr6:72418120|72430205 were significantly up-regulated in the BCP group compared with that of the sham group (**Fig. 5A**; *P* < 0.05), consistent with the previous sequencing results. On the contrary, chr18:69943105|69944476 and chr5:167516581|167558250 were significantly down-regulated, also consistent with the sequencing results. In addition, circular primers (divergent primers) and linear primers (convergent primer) were designed as previously reported for chr6:72418120|72430205, chr20:7561057|7573740, chr18:69943105|69944476, and chr5:167516581|167558250 to carry out PCR analysis. The PCR amplification was analyzed by agarose gel electrophoresis and Sanger sequencing. The results indicated that the four circRNAs exhibited bands of expected size in the cDNA of the BCP group but not in the gDNA analysis (**Fig. 5B,C**). Meanwhile, the back-spliced junction site was validated by Sanger sequencing. Based on the expression level and sequence length, we selected chr6:72418120|72430205, which corresponds to Strn3, for further analysis.

### Knockdown of circStrn3 may promote Walker 256 cell apoptosis

**Figure 6:**
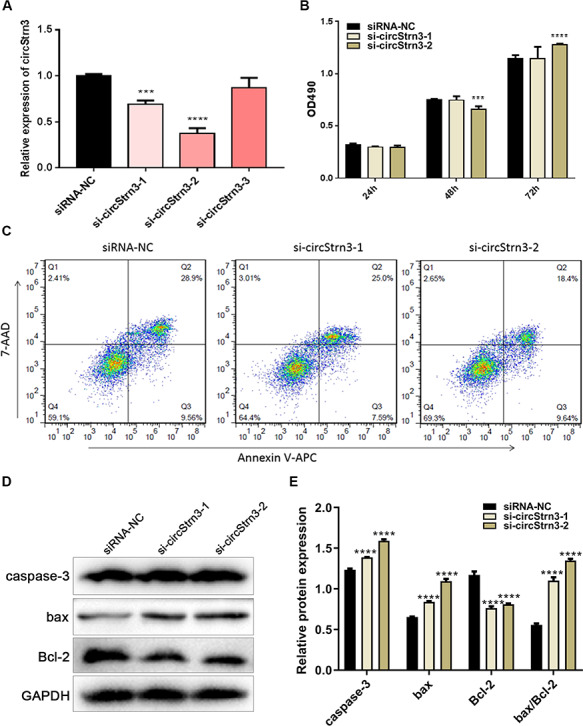
**Knockdown of circStrn3 may promote Walker 256 cell apoptosis** (A) circStrn3 expression in Walker 256 cells with circStrn3 knockdown. (B) CCK-8 assay was used to measure Walker 256 cell proliferation upon knockdown of circStrn3. (C) Flow cytometry analysis of Walker 256 cells upon knockdown of circStrn3. (D) Western blot analysis of Walker 256 cells upon knockdown of circStrn3. (E) Semi-quantitative results of graph D. ^***^P < 0.001 and ^****^P < 0.0001.

To further investigate the function of chr6:72418120|72430205 (circStrn3), we designed three siRNAs to knockdown the expression of circStrn3 in Walker 256 cells. si-circStrn3-1 and si-circStrn3-2 could effectively inhibit circStrn3 expression in Walker 256 cells (**Fig. 6A**; *P* < 0.05), and si-circStrn3-3 had little effect. Based on the above result, we chose si-circStrn3-1 and si-circStrn3-2 for further studies. Walker 256 cells were transfected with si-circStrn3-1 or si-circStrn3-2, and then cell proliferation was detected by CCK-8 assay. **Figure 6B** showed that cell proliferation was decreased 48 h after transfection with si-circStrn3-2 but increased 72 h after transfection. At the same time, cell proliferation did not change after transfection with si-circStrn3-1. To analyze circStrn3-related cell apoptosis, we employed flow cytometry to examine Walker 256 cells transfected with si-circStrn3-1 or si-circStrn3-2. **Figure 6C** showed that 48 h after transfection with si-circStrn3-1, early apoptosis was decreased in Walker 256 cells (7.59%) compared with siRNA-NC (9.56%). Meanwhile, 48 h after transfection with si-circStrn3-2, the number of early apoptotic cells was higher than that in the siRNA-NC-transfected group. To further analyze the relationship between circStrn3 and apoptosis, western blot analysis was used to detect the expression levels of apoptosis-related proteins, including caspase-3, bax, and Bcl-2, in Walker 256 cells transfected with si-circStrn3-2 or si-circStrn3-1. The results showed that knockdown of circStrn3 significantly increased the protein levels of caspase-3 and bax and inhibited the protein level of Bcl-2 (**Fig. 6D,E**). Therefore, we speculated that circStrn3 may promote Walker 256 cell apoptosis.

## Discussion

BCP is the most common type of cancer pain and is also the most severe and difficult to control. It is often manifested as continuous background pain, breakthrough pain, and allodynia [35,36]. BCP pathogenesis may include inflammatory and neuropathic pain. However, it is not the same as inflammatory pain and/or neuropathic pain [8]. A previous study showed that plasticity changes in spinal dorsal horn neurons are important causes of cancer pain formation and maintenance [37]. However, detailed molecular mechanisms of these manifestations are still unclear.

The lack of a suitable animal model of cancer pain hindered the progress in dissecting the mechanism of cancer pain. However, in recent years, the establishment of mouse femur, calcaneus, and humerus and rat sacral cancer pain models has greatly promoted the understanding of cancer pain [38–40]. Walker 256 breast cancer cells possess strong tumorigenicity, bone invasiveness, and reproduction. Therefore, these cells have been widely used to establish rat cancer pain model [41]. Female rats are susceptible to Walker 256 breast cancer cells, which minimizes the impact of sexual maturation on pain experiments [42]. The evaluation of the cancer pain model depends mainly on the model’s simulation of clinical cancer pain [43]. Herein, we found that rats in the BCP group began to lose weight 9 days after implantation of the left tibia tumor, which was consistent with results reported by Muta *et al*. [44]. The PWT score of the BCP group is gradually decreased from 7 days after tumor implantation. Bone destruction and pain are gradually worsened in patients with bone metastases. Meanwhile, the patients often have an outbreak of pain, including spontaneous rest pain and incident pain [35]. Behavioral and bone damages are important indicators for evaluating the success of the BCP model [45]. H&E staining of the left tibia in the BCP group showed that the trabecular bone of the tibia was damaged 21 days after tumor implantation. The cortical bone was also severely damaged. Tumor cells filled the marrow cavity. All these results indicated that the implantation of Walker 256 tumor cells at the upper end of the tibia of SD rats could successfully establish a model of tibial cancer pain.

circRNAs are highly conserved and stable in mammals. Therefore, they have the potential to be ideal biomarkers for the diagnosis of cancer [46,47]. Li *et al*. [48] found that hsa_circ_002059 was significantly down-regulated in gastric cancer tissues. The expression level of this molecule is correlated with gastric cancer cell metastasis, TNM stage, sex, and age. Meanwhile, a previous study suggested that ciRS-7 was differentially expressed in liver cancer, cervical cancer, and malignant glioma [49]. ciRS-7 can down-regulate miR-7 expression and abolish its inhibitory effect on the IGF1R gene. Therefore, ciRS-7 can further induce cancer cell proliferation, invasion, and metastasis [49]. Furthermore, there is a close relationship between circITCH and esophageal cancer, colorectal cancer, and lung cancer [50]. Recently, the host gene PVTI of circPVT1 was shown to be involved in the development of cancer via regulating the protein stability of the proto-oncogene c-myc [51,52]. The hsa_circ_001988 is reduced in cancer tissues and is associated with the extent of tumor cell differentiation and prognosis [53]. Therefore, circRNAs can be used as novel biological markers for disease diagnosis and drug development.

In this study, we screened DECs between the BCP group and control group. KEGG analysis showed that MAPK was significantly enriched. The MAPK cascade is an important signal transduction system, and the MAPK signaling pathway is involved in the modulation of pain transmission, including neuropathic pain and inflammatory pain [54–56]. The three major signaling pathways, i.e. p38, ERK and c-Jun, of the MAPK family are distributed in the spinal cord. Particularly, the ERK signaling pathway is distributed in neurons, microglia, and astrocytes [57]. The Jun signaling pathway is mainly distributed in astrocytes, with minimal distribution in neurons and microglia [58]. The p38 signaling pathway is mainly found in microglia [59]. Glial cells play an important role in chronic pain [60]. Meanwhile, microglia and astrocytes have various degrees of activation at different stages of inflammatory pain and neuralgia [61]. Furthermore, the PI3K–Akt signaling was also found to be enriched in this study. The PI3K–Akt–mTOR signaling pathway is one of the bridges linking signal transduction and cellular response. This pathway is involved in many physiological processes, such as cell metabolism, proliferation, differentiation, and apoptosis [62]. In BCP research, Han *et al*. [62] showed that formaldehyde can activate TRPV1 through the PI3K signaling pathway, which participates in the formation of BCP. Meanwhile, Shih *et al*. [63] established a BCP model using prostate cancer cells and demonstrated a time-dependent increase in pmTOR and its downstream effector molecule p-p70S6K in the spinal dorsal horn (L4-5) on the ipsilateral side of the carcinogenesis with the development of cancer pain, suggesting an important role of the PI3K–Akt–mTOR signaling pathway in BCP.

We further found that circStrn3 was differentially expressed between the BCP group and sham group. Functional study suggested that knockdown of circStrn3 could effectively affect cancer cell apoptosis and proliferation, indicating that circStrn3 might be a potential biomarker for BCP. Through the ceRNA network, circStrn3 could regulate rno-miR-9a-5p to influence Nfkb1, and Nfkb1 was found to be highly expressed in BCP rats. Pathological activation of Nfkb1 may be involved in the development of various inflammatory and rheumatic diseases, such as osteoarthritis and rheumatoid arthritis in the bone [63]. Previous studies have shown that the expression of NF-κB is significantly increased in the spinal cord tissue of a rat BCP model, suggesting that activation of the NF-κB pathway is associated with BCP development [64]. As an important transcription factor in cells, NF-κB is widely distributed in neuronal and glial cells. This protein can initiate transcription of various cytokine genes in polymorphonuclear leukocytes, which may induce the inflammatory response. A previous study suggested that the inflammatory response is an important change in the pathophysiological process of cancer pain. Meanwhile, a variety of inflammatory cytokines, including TNF-α and IL-6, play a pivotal regulatory role in the process of chronic pain [65,66]. The external stimulus signal activates IκB kinase, which phosphorylates the IκB protein. Phosphorylated IκB can be degraded by ubiquitination. Therefore, NF-κB may initiate the expression of the corresponding target genes by translocating into the nucleus [67]. Based on the above evidence, we speculated that Nfkb1 expression regulated indirectly by circStrn3 may be closely associated with BCP.

In conclusion, circStrn3 is a potential diagnostic and therapeutic target for BCP. The role of circStrn3 in cancer pain still warrants further study. Additionally, circRNAs may play critical roles in disease diagnosis, treatment and drug development.

## Supplementary Data

Supplementary data is available at *Acta Biochimica et Biophysica Sinica* online.

## Funding

This work was supported by the grants from the Medical Science and Technology Research Foundation of Guangdong Province (No. A2019045), the Scientific Research Foundation of Foshan City, Guangdong Province (No. 1920001000687), the 2018 Distinguished Youth Talent Fund Project of the First Medical Science Center of Foshan City, and the Key Specialist Project of Clinical Medicine of Foshan City (No. FSZDZK135049).

## Supplementary Material

Suppl_data_gmaa018
